# The complete chloroplast genome sequence of *Spathodea campanulata*

**DOI:** 10.1080/23802359.2019.1674710

**Published:** 2019-10-11

**Authors:** Yi Wang, Xiaolong Yuan, Yunqing Li, Jinfeng Zhang

**Affiliations:** Laboratory of Forest Plant Cultivation and Utilization, Yunnan Academy of Forestry, Kunming, Yunnan, People's Republic of China

**Keywords:** *Spathodea campanulata*, chloroplast, Illumina sequencing, phylogenetic analysis

## Abstract

The first complete chloroplast genome (cpDNA) sequence of *Spathodea campanulata* was determined from Illumina HiSeq pair-end sequencing data in this study. The cpDNA is 158,001 bp in length, contains a large single-copy region (LSC) of 84,923 bp and a small single-copy region (SSC) of 12,566 bp, which were separated by a pair of inverted repeat (IR) regions of 30,256 bp. The genome contains 131 genes, including 86 protein-coding genes, 8 ribosomal RNA genes, and 37 transfer RNA genes. The overall GC content of the whole genome is 37.8% and the corresponding values of the LSC, SSC, and IR regions are 35.9, 32.6, and 41.7%, respectively. Further, phylogenomic analysis showed that *S. campanulata* clustered in a unique clade in family *Bignoniaceae*.

*Spathodea campanulata* is the species of the genus Spathodea within the family Bignoniaceae, native from equatorial Africa (Pianaro et al. 2007). It is often used in gardening in tropical and subtropical areas, including Fujian, Taiwan, Yunnan province of China (Yang et al. 2014). *Spathodea campanulatais* was widely used as traditional medicine employed to control epilepsy in Nigeria (Ilodigwe et al. 2010). The stem bark extract of *S. companulata* showed antimalarial and anti-HIV activity (Amusan et al. 1996; Niyonzima et al. 1999). The extract of *S. companulata* also showed antimicrobial and anti-proliferative activities (Teinkela et al. 2016). Therefore, *S. companulata* has huge medicinal value. However, there have been no genomic studies on *S. campanulata*.

Herein, we reported and characterized the complete *S. campanulata* plastid genome (MN106255). One *S. campanulata* individual (specimen number: 201812029) was collected from Puwen, Yunnan Province of China (23°76′13″ N, 101°28′17″ E). The specimen is stored at Yunnan Academy of Forestry Herbarium, Kunming, China and the accession number is YAFH0012768. DNA was extracted from its fresh leaves using DNA Plantzol Reagent (Invitrogen, Carlsbad, CA, USA).

Paired-end reads were sequenced by using Illumina HiSeq system (Illumina, San Diego, CA, USA). In total, about 30.4 million high-quality clean reads were generated with adaptors trimmed. Aligning, assembly, and annotation were conducted by CLC de novo assembler (CLC Bio, Aarhus, Denmark), BLAST, GeSeq (Tillich et al. 2017), and GENEIOUS v 11.0.5 (Biomatters Ltd, Auckland, New Zealand). To confirm the phylogenetic position of *S. campanulata*, other eight species of family *Bignoniaceae* from NCBI were aligned using MAFFT v.7 (Katoh and Standley 2013) and maximum likelihood (ML) bootstrap analysis was conducted using RAxML (Stamatakis 2006); bootstrap probability values were calculated from 1000 replicates. *Andrographis paniculata* (KF150644) and *Echinacanthus lofouensis* (MF490441) were served as the out-group.

The complete *S. campanulata* plastid genome is a circular DNA molecule with the length of 158,001 bp, contains a large single-copy region (LSC) of 84,923 bp and a small single-copy region (SSC) of 12,566 bp, which were separated by a pair of inverted repeat (IR) regions of 30,256 bp. The overall GC content of the whole genome is 37.8%, and the corresponding values of the LSC, SSC, and IR regions are 35.9, 32.6, and 41.7%, respectively. The plastid genome contained 131 genes, including 86 protein-coding genes, 8 ribosomal RNA genes, and 37 transfer RNA genes. Phylogenetic analysis showed that *S. campanulata* clustered in a unique clade in family *Bignoniaceae* (Figure 1). The determination of the complete plastid genome sequences provided new molecular data to illuminate the *Bignoniaceae* evolution.

**Figure 1.**
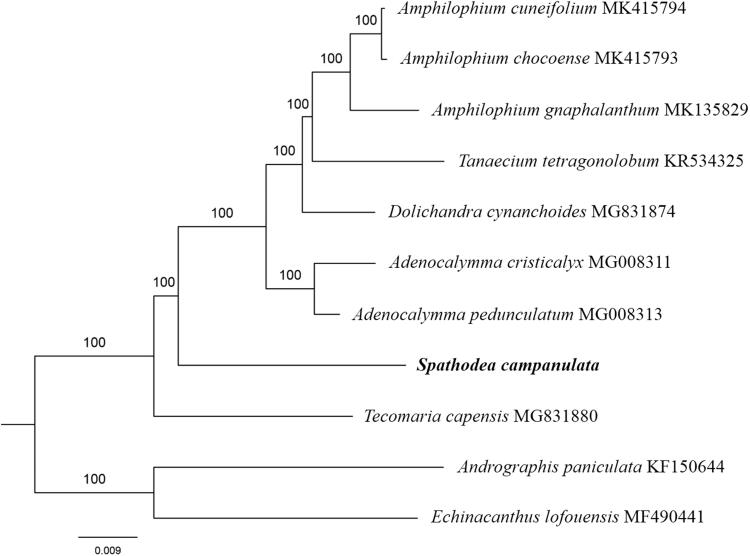
The maximum-likelihood tree based on the 9 chloroplast genomes of *Bignoniaceae*. The bootstrap value based on 1000 replicates is shown on each node.
